# Habitat Selection in a Rocky Landscape: Experimentally Decoupling the Influence of Retreat Site Attributes from That of Landscape Features

**DOI:** 10.1371/journal.pone.0037982

**Published:** 2012-06-12

**Authors:** Benjamin M. Croak, David A. Pike, Jonathan K. Webb, Richard Shine

**Affiliations:** 1 School of Biological Sciences, University of Sydney, Sydney, New South Wales, Australia; 2 School of Marine and Tropical Biology, James Cook University, Townsville, Queensland, Australia; 3 School of the Environment, University of Technology Sydney, Sydney, New South Wales, Australia; Australian Wildlife Conservancy, Australia

## Abstract

Organisms selecting retreat sites may evaluate not only the quality of the specific shelter, but also the proximity of that site to resources in the surrounding area. Distinguishing between habitat selection at these two spatial scales is complicated by co-variation among microhabitat factors (i.e., the attributes of individual retreat sites often correlate with their proximity to landscape features). Disentangling this co-variation may facilitate the restoration or conservation of threatened systems. To experimentally examine the role of landscape attributes in determining retreat-site quality for saxicolous ectotherms, we deployed 198 identical artificial rocks in open (sun-exposed) sites on sandstone outcrops in southeastern Australia, and recorded faunal usage of those retreat sites over the next 29 months. Several landscape-scale attributes were associated with occupancy of experimental rocks, but different features were important for different species. For example, endangered broad-headed snakes (*Hoplocephalus bungaroides*) preferred retreat sites close to cliff edges, flat rock spiders (*Hemicloea major*) preferred small outcrops, and velvet geckos (*Oedura lesueurii*) preferred rocks close to the cliff edge with higher-than-average sun exposure. Standardized retreat sites can provide robust experimental data on the effects of landscape-scale attributes on retreat site selection, revealing interspecific divergences among sympatric taxa that use similar habitats.

## Introduction

Many animals spend long periods (on a diel cycle, and/or seasonally) sheltered within retreat sites and the choice of retreat site may influence organismal fitness [1]–[3]. Thus, it is not surprising that both field-survey and experimental studies reveal strongly non-random selection of retreat sites by animals, based on a diverse array of biotic and abiotic cues. For example, the frog *Phrynobatrachus guineensis* breeds in tree hollows and selects nesting sites that contain conspecifics (thereby reducing the chance of predation) and suitable hydric regimes [4]. Common Brushtail possums (*Trichosurus vulpecula*) living in woodland habitat select tree-hollows high above the ground that provide protection from predators, a buffer against environmental extremes, and favorable temperatures [5]. Many ectotherms select retreat sites based on thermal regimes [6]–[9], scent cues from other species [10]–[12], and/or the three-dimensional structure of the retreat site itself [13].

Most research on retreat site selection has focused on the attributes of individual retreat sites. However, habitat selection by animals also involves criteria that relate to a much larger spatial scale. Many species are restricted to distinctive macrohabitats (e.g., rocky areas, thick forests, and the like) so that to understand habitat selection, we need to gather data at a variety of spatial scales [14], [15]. For example, red foxes (*Vulpes vulpes*) create dens at non-random sites at both small spatial scales (i.e., on slopes that provide stable soils) and at large spatial scales (i.e., close to foraging sites and water bodies [16]). By analogy, people buying homes are influenced not only by the specific features of the house, but also by the resources accessible from that site. Indeed, the latter often may be more important (as suggested by the real-estate agent’s adage that the three most important factors in house desirability are “location, location, location”).

One important challenge to understanding habitat selection is the effect of co-variation of features across multiple spatial scales. For example, the availability of loose surface rocks (potential retreat sites) often will be higher close to a large rock outcrop, so that a tendency for animals to shelter under rocks found close to an outcrop might reflect either features of the specific shelter (because more choice is available closer to the outcrop), or the proximity of the outcrop itself (and hence, access to resources such as food, water, or escape from predators). Similarly, proximity to woodland might affect both shading levels experienced by a given rock (and thus, thermal regimes within the retreat site) as well as effects of woodland proximity *per se* (such as the distances to resources [e.g., food, water, and nesting sites] restricted to that habitat type). Such co-variation makes it difficult to distinguish criteria for habitat selection at a landscape scale as opposed to the level of the individual retreat site.

To experimentally test the causal role of habitat attributes in retreat-site selection at larger spatial scales, we need to standardize the attributes of individual retreat sites, in order to minimize variance in habitat-selection resulting from faunal preferences at that smaller spatial scale. Artificially-created retreat sites (such as nest boxes provided for bird breeding [17]) are well suited to this purpose, enabling researchers to investigate faunal responses to habitat-scale factors by creating near-identical retreat sites in a range of locations.

The system we have investigated involves crevice use by nocturnal rock-dwelling animals and is part of a long-term study of this system. Previous research shows that many nocturnal rock-dwelling species utilize both structural and biotic cues to select diurnal retreat sites. These include physical space configurations [13], temperatures [7], [18]–[20], moisture levels [21], and scent cues from predators [20], [22], [23], prey [11], [12], [24], and conspecifics [22]. At a large spatial scale, GIS studies have documented significant associations between species distributions and climatic variables [25] and overall landscape features (e.g., availability of sandstone rocks [26]). We designed our study to fill the gap between these two spatial levels of analysis, by investigating habitat selection at intermediate spatial scales. To do so, we constructed and deployed artificial rocks that are identical to each other in size and crevice structure (thereby controlling for factors intrinsic to the retreat site). We also quantified the location of the rock in terms of several habitat variables, and tested how faunal use relates to these variables.

**Figure 1:**
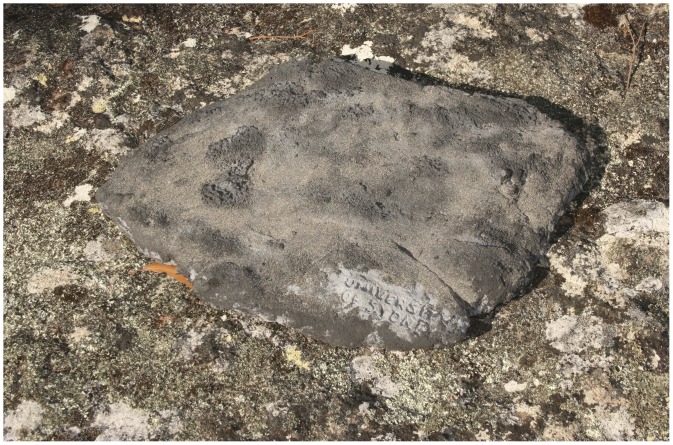
An artificial rock on site. Artificial rocks were designed to provide crevices with attributes preferred by saxicolous reptiles. All rocks were placed on flat ground to provide crevices 4 to 11 mm high, in areas with open canopies overhead and on the western side of the outcrops to allow relatively high sun exposure (and thus, favorable thermal regimes).

## Materials and Methods

### Ethics Statement

Permits were provided specifically for this project by the University of Sydney Animal Care and Ethics Committee (permit L04/12-2008/3/4927).

**Table 1:** Details of generalized linear mixed models comparing the attributes of used *versus* unused artificial rocks by species.

	Broad-headed snake	Small-eyed snake	Velvet gecko	Red-throated skink	Wall Skink	Copper-tailed skink	Flat rock spider
Number of artificial rocks colonized (%)	18 (9.09)	13 (6.57)	165 (83.33)	28 (14.14)	105 (53.03)	20 (10.1)	165 (83.33)
Radiation (w_i_)	**0.66±0.45 (0.54)**	−0.10±0.32 (0.28)	0.10±0.27 (0.28)	−0.24±0.23 (0.40)	0.22±0.21 (0.39)	0.22±0.26 (0.33)	0.19±0.20 (0.35)
Outcrop area (w_i_)	−1.14±1.13 (0.39)	−0.02±0.96 (0.43)	**0.63±0.57 (0.50)**	0.03±0.26 (0.26)	0.07±0.17 (0.28)	0.25±0.36 (0.30)	−**0.29±0.18 (0.60)**
Nearest natural rock area (w_i_)	−0.06±0.02 (0.31)	0.27±0.28 (0.36)	0.33±0.26 (0.47)	**0.43±0.19 (0.81)**	−0.22±0.17 (0.45)	−0.30±0.30 (0.38)	0.11±0.12 (0.29)
Distance to west-facing cliff (w_i_)	−**1.82±0.83 (0.94)**	−**1.10±0.68 (0.72)**	−**0.42±0.26 (0.62)**	−0.16±0.31 (0.30)	−0.13±0.18 (0.32)	−**0.52±0.26 (0.53)**	−0.16±0.22 (0.35)
Distance to woodland (w_i_)	0.16±0.34 (0.29)	−0.05±0.36 (0.28)	0.32±0.27 (0.44)	−**0.37±0.26 (0.51)**	**0.27±0.17 (0.56)**	−**1.09±0.42 (0.98)**	−0.19±0.20 (0.36)
Distance to nearest natural rock (w_i_)	0.25±0.29 (0.34)	0.17±0.31 (0.29)	−**0.44±0.26 (0.58)**	−0.05±0.24 (0.26)	0.03±0.17 (0.26)	**0.58±0.26 (0.81)**	−0.18±0.18 (0.36)
Distance to leaf litter (w_i_)	**0.38±0.26 (0.51)**	−**1.00±0.58 (0.77)**	**0.52±0.33 (0.61)**	−**0.41±0.28 (0.54)**	**0.54±0.20 (0.96)**	−0.43±0.40 (0.41)	−0.07±0.20 (0.28)
Distance to nearest crevice (w_i_)	−0.20±0.37 (0.30)	**0.13±0.42 (0.76)**	0.01±0.26 (0.27)	−0.26±0.29 (0.36)	−0.12±0.17 (0.31)	−0.53±0.42 (0.36)	0.22±0.24 (0.36)

Sample sizes, average parameter estimates, standard errors of these estimates, and sum of Akaike weights (w_i_) for explanatory variables derived for all combinations of the generalized linear models comparing the attributes of used artificial rocks *versus* those not known to have been used by seven different wildlife species on sandstone outcrops in south-eastern Australia. Boldface values have a high w_i_ value, indicating high importance.

### Study Sites, Rock Placement, and Sampling

We placed 198 artificial rocks on flat areas of two sandstone plateaus near Nowra, south-eastern New South Wales, Australia. The rocks were created to restore anthropogenically-degraded habitat that supports a unique assemblage of specialized fauna. The rocks were designed to create crevices that were structurally and thermally similar to natural rocks used by these target faunal assemblages [13], [27]. We deployed these rocks non-randomly, based on previous studies of the target faunal groups. That is, we placed them on flat ground in open areas close to the outcrop edges [13], [27]. These sites consist of relatively small open clearings within eucalypt forest, close to steep cliffs (up to 50 m high) that prevent trees from shading the rocky areas along the cliff edge [28]. The area contains an endangered snake species, the broad-headed snake (*Hoplocephalus bungaroides*), its major lizard prey, the velvet gecko (*Oedura lesueurii*), and a wide range of other ectotherms [28], [29]. Thus, the artificial rocks were placed out non-randomly based on our knowledge of the ecology of these species; that is, we placed artificial rocks on the western/north-western side of the plateaus and in areas with relatively open canopies (i.e., areas that receive high levels of incident radiation; see [19], [30] (see Figure 1). Incident radiation determines the thermal regime experienced in the retreat site created between the rocks and the substrate [19]. We avoided any sites with soil or leaf-litter substrate, or that were shaded by overhanging trees [28]. The artificial rocks were identical in size, shape, thickness, and coloration (broadly rectangular, 550×385 mm, with each rock ranging in thickness from 27–65 mm [13]; Figure 1) and differed only in the number of entrance holes. Half of the artificial rocks (N = 99) contained four entrance holes, whereas the other half (N = 99) contained two entrance holes; because these variables do not appear to influence reptile use [27], we treated all rocks as identical replicates for analyses in the current study. Each artificial rock was constructed and deployed to create similar thermal regimes, crevice structure and aspect exposure, all of which influence the use of these artificial rocks by habitat specific fauna [13], [27]. Because we controlled for factors that are known to influence retreat site use in these taxa (crevice configuration [13] and aspect, canopy cover and resultant incident radiation; see above), any non-random patterns of retreat site selection should reflect other, previously unrecognized variables.

**Figure 2:**
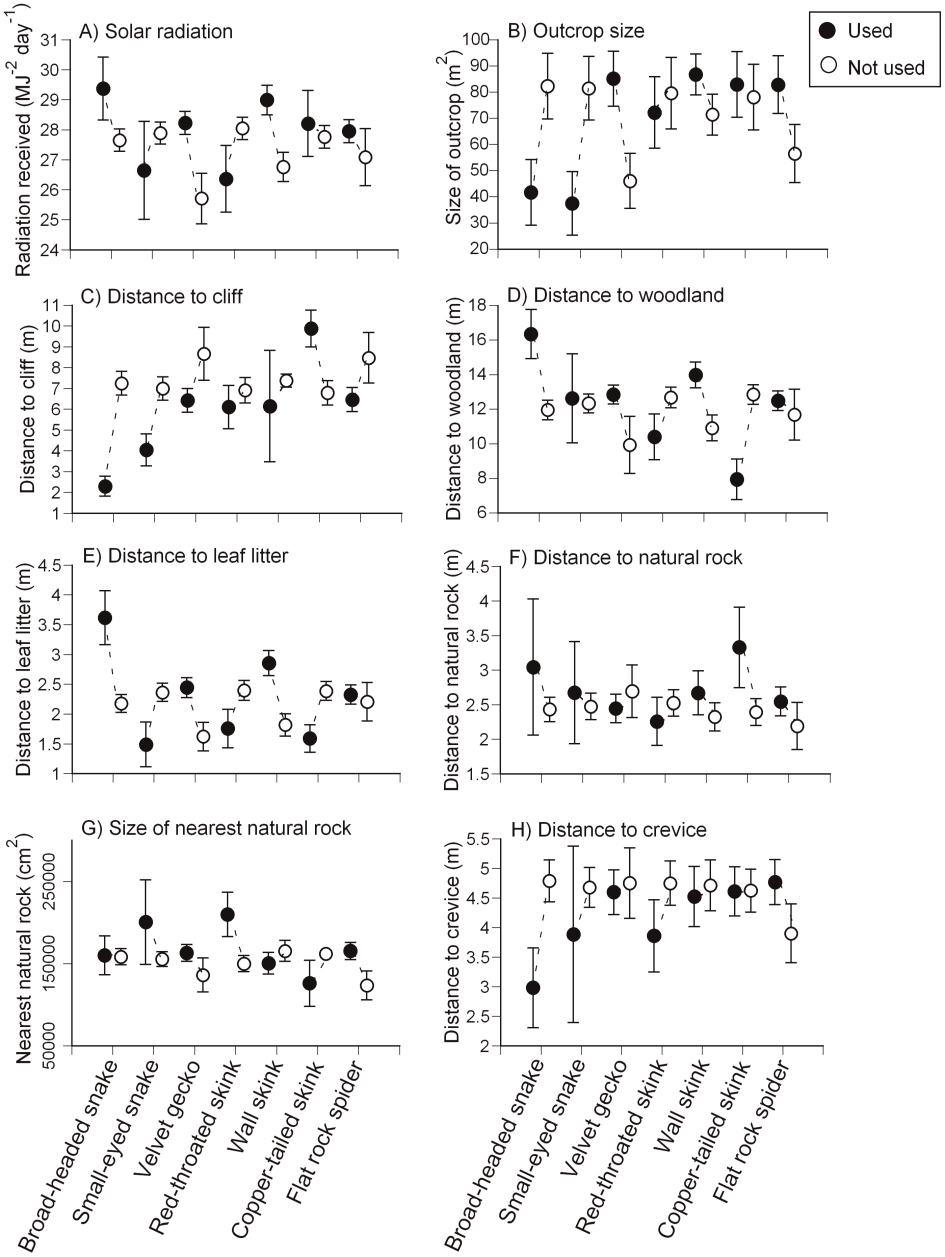
Means and standard errors of habitat variables associated with artificial rocks either used or not used by seven saxicolous wildlife species. “Used” rocks were those where we found the species sheltering in the crevice formed between the artificial rock and the underlying rock substrate. These values are based on measurements of 198 identical artificial rocks deployed across the landscape, and the use of those rocks by fauna over 29 months, from August 2007–December 2009.

We deployed the artificial rocks late in the austral winter (August 2007) and monitored their use by reptiles and invertebrates every two weeks from August to November 2007 (N = 8 sampling sessions) and on a monthly basis thereafter (from December 2007 to December 2009, N = 25 sessions; total N = 33 sampling sessions spanning 29 months). During sampling, we turned all rocks and captured, identified, marked, and released any animals using the crevice formed between the rock and the underlying substrate. All rocks were sampled on all sampling occasions. For analysis, we treated any rock that harbored a given species on any of the 33 sampling trips as used by that species. We conducted analyses of used *versus* unused rocks for each species, and also examined frequency of use within the subset of artificial rocks known to have been used by each taxon.

**Table 2:** Details of generalized linear mixed models predicting the frequency of use of artificial rocks by species.

	Broad-headed snake	Small-eyed snake	Velvet gecko	Red-throated skink	Wall Skink	Copper-tailed skink	Flat rock spider
Radiation (w_i_)	–	−0.05±0.27 (0.27)	**0.12±0.04 (0.99)**	−0.24±0.23 (0.40)	0.10±0.09 (0.37)	0.28±0.21 (0.46)	−**0.17±0.04 (1.00)**
Outcrop area (w_i_)	–	−**1.35±0.94 (0.60)**	**0.11±0.04 (0.95)**	0.03±0.26 (0.26)	**0.14±0.09 (0.57)**	0.27±0.33 (0.31)	−0.05±0.04 (0.45)
Nearest natural rock area (w_i_)	–	−0.01±0.26 (0.26)	0.02±0.03 (0.32)	**0.43±0.19 (0.81)**	−0.00±0.06 (0.25)	−0.18±0.23 (0.33)	0.03±0.03 (0.38)
Distance to west-facing cliff (w_i_)	–	−**0.73±0.49 (0.60)**	−**0.33±0.05 (1.00)**	−0.24±0.22 (0.36)	−**0.23±0.12 (0.80)**	−**0.60±0.37 (0.73)**	0.04±0.04 (0.37)
Distance to woodland (w_i_)	–	0.05±0.27 (0.28)	−0.01±0.04 (0.27)	−**0.37±0.26 (0.51)**	**0.26±0.08 (0.99)**	−**1.06±0.35 (1.00)**	0.03±0.04 (0.34)
Distance to nearest natural rock (w_i_)	–	−0.06±0.31 (0.26)	−**0.04±0.03 (0.51)**	−0.05±0.24 (0.26)	−0.05±0.07 (0.31)	**0.51±0.19 (0.89)**	−**0.10±0.04 (0.93)**
Distance to leaf litter (w_i_)	–	−**0.89±0.48 (0.79)**	0.01±0.03 (0.28)	−**0.41±0.29 (0.54)**	**0.20±0.07 (0.95)**	−**0.49±0.35 (0.54)**	**0.06±0.03 (0.63)**
Distance to nearest crevice (w_i_)	–	−0.43±0.46 (0.39)	−0.04±0.04 (0.39)	−0.26±0.29 (0.36)	**0.20±0.07 (0.99)**	−0.23±0.35 (0.32)	**0.08±0.03 (0.78)**

Average parameter estimates, standard errors of these estimates, and sum of Akaike weights (w_i_) for explanatory variables derived for all combinations of the generalized linear models predicting the frequency of usage of artificial rocks by six different wildlife species on sandstone outcrops in south-eastern Australia. Rocks never recorded as being used by a given species are omitted from these analyses. Bold values have a high w_i_ value, indicating high importance.

### Rock Attributes

For each artificial rock, we measured the following environmental factors: the incident radiation received by each rock (MJ/m^2^ per day: quantified by taking 180° hemispherical photographs of the forest canopy directly above each artificial rock and importing them into Gap Light Analyzer software (GLA). Incident radiation is calculated from canopy cover determined by GLA and inputted location and day length data [31], [32]); distance to the closest west or north-west facing cliff (m); distance to adjacent woodland (m); distance to the nearest natural rock large enough to house our focal species (m) and the size (length × width; cm^2^) of that nearest rock; distance to leaf-litter (m); distance to nearest rock crevice large enough to house our focal species (m); and the size of the contiguous bare rock outcrop on which the artificial rock was located (outcrop area; length × width, m^2^). We recorded linear dimensions using a tape measure (to 0.5 cm).

### Data Analysis

We used the statistical package R (2.10.0) for all analyses [33]. Because habitat variables are often correlated at different habitat scales, we used Spearman’s rank correlation tests to assess whether the habitat variables that we measured were significantly correlated with one another. No variables were significantly correlated (all p>0.05), so we included them all in the analyses [34]. To allow comparison of model parameter estimates, we standardized all variables to a mean of zero and a standard deviation of one. To compare factors that influenced rock usage by each species, we used univariate generalized linear mixed models (GLMM) with the binomial family (link function type = logit) and ranked the models using a corrected Akaike’s information criteria (AIC_c_ [35]), with site as the random factor. We also investigated factors influencing the relative frequency of use of artificial rocks (among those used at least once by that species, thus omitting data for rocks that were never used) by developing univariate generalized linear models using the Poisson family (link function type = Poisson) and ranking the models using AIC_c_. For both analyses, we used a model averaging approach to account for model and parameter uncertainty [35]. We developed alternative models from all linear combinations of the explanatory variables, ranked these by their AIC_c_ values and obtained the Akaike weight for each model [35]. Magnitude and direction of the effect of a variable were calculated from model-averaged parameter estimates, which we obtained by using the mean of the coefficient estimates of all models weighted by the Akaike weight. We also assessed the relative importance of individual variables for each target species by summing the Akaike weights from all model combinations where the variable occurred, then ranking the variables according to their Akaike weight, with larger values indicating greater importance [35].

## Results

AIC rankings of the results from our GLMM analyses showed that our seven study species; broad-headed snakes (*Hoplocephalus bungaroides*), small-eyed snakes (*Cryptophis nigrescens*), velvet geckos (*Oedura lesueurii*), red-throated skinks (*Acritoscincus platynotum*), copper-tailed skinks (*Ctenotus taeniolatus*), wall skinks (*Cryptoblepharus pulcher*) and flat rock spiders (*Hemicloea major*) used rocks non-randomly with respect to intermediate-scale habitat attributes. We focus below on those with high importance based on the sum of Akaike weights (Table 1).

### Used *versus* Unused Artificial Rocks

All seven of the species that we studied were recorded often enough for us to conduct robust comparisons between the habitat attributes surrounding used *versus* unused rocks (Table 1). Velvet geckos showed non-random rock use with respect to four variables, red-throated skinks, copper-tailed skinks, small-eyed snakes and broad-headed snakes showed non-random rock use with respect to three variables (Table 1, Figure 2), wall skinks responded to two variables, and flat-rock spiders responded to a single variable (Table 1, Figure 2).

Velvet geckos appeared to base retreat-site selection on more habitat variables than did any other species. The geckos chose rocks on large outcrops, close to the cliff edge, far from leaf litter and close to natural rocks (Table 1, Figure 2B, 2C, 2E, 2F). Of the four species that responded to three variables, three were affected by the distance of artificial rocks from nearby leaf-litter. Broad-headed snakes chose rocks far from leaf litter, whereas small-eyed snakes and red-throated skinks chose rocks close to leaf litter (Table 1, Figure 2E). Small-eyed snakes and broad-headed snakes also both chose rocks close to the cliff (Table 1, Figure 2C), but differed in other criteria. Broad-headed snakes chose rocks that received higher than average solar radiation (Table 1, Figure 2A) and small-eyed snakes chose rocks located far from crevices (Table 1, Figure 2H). Red-throated skinks selected rocks close to woodland and near large natural rocks (Table 1, Figure 2D, 2G). Copper-tailed skinks showed a preference for artificial rocks close to the cliff and woodland yet far from other rocks (Table 1, Figure 2C, 2D, 2F). Wall skinks chose artificial rocks far from woodland and leaf litter (Table 1, Figure 2D, 2E). Finally, flat rock spiders were most common under artificial rocks located on smaller outcrops (Table 1, Figure 2B).

### Frequency of Artificial Rock Usage

Broad-headed snakes used individual artificial rocks too infrequently for statistical analysis, because this endangered species is too rare to generate suitable sample sizes. However, the remaining six species showed strong patterns.

Wall skinks commonly used artificial rocks that were influenced by five habitat variables: rocks on large outcrops, close to the cliff edge, far from crevices, leaf litter and woodland (Table 2). Three of the remaining five species (velvet geckos, copper-tailed skinks and flat rock spiders) commonly used artificial rocks that were distinctive in terms of four habitat variables. Rock usage by these species was influenced by distance to the nearest natural rock (Table 2); velvet geckos and flat rock spiders preferred artificial rocks close to natural rocks, whereas copper-tailed skinks showed the opposite preference (Table 2). Velvet geckos and copper-tailed skinks both preferred artificial rocks close to the cliff, but differed in other respects. The geckos were found most often beneath artificial rocks exposed to higher-than-average radiation, and located on large outcrops (Table 2). In contrast, copper-tailed skinks repeatedly used artificial rocks that were close to leaf litter and adjacent woodland (Table 2). As well as preferring artificial rocks that were close to natural ones, flat rock spiders repeatedly used rocks that received less-than-average radiation exposure and that were located far from leaf litter and crevices (Table 2). Rock use by the remaining two species, small-eyed snakes and red-throated skinks, was influenced by three variables. Both species preferred rocks close to leaf litter, but small-eyed snakes selected rocks on small outcrops close to the cliff edge (Table 2), whereas red-throated skinks used artificial rocks close to woodland and large natural rocks (Table 2).

## Discussion

By standardizing three major aspects of individual retreat sites that influence thermal regimes (rock size and thickness, three-dimensional crevice structure beneath the rock, and canopy openness [7], [13], [19], [30]), we showed that landscape-scale features influence habitat selection by most of the rock-dwelling species that we studied (Tables 1 and 2; Figure 2). Importantly, each of the seven species showed different patterns of spatial association with landscape features. Below, we first consider the nature of (and possible causes for) such patterns, before considering the broader implications of our results.

### Used *versus* Unused Artificial Rocks

Broad-headed snakes selected artificial rocks exposed to high levels of incident radiation (Table 1, Figure 2A). This result supports previous work (based on selection of natural rocks by snakes and lizards) that has identified thermal cues as important in diurnal retreat site selection for many nocturnal saxicolous reptile species [7], [9], [18], [19]. Previous experiments have also shown that thermal cues influence retreat site selection in four species whose spatial distributions were not strongly associated with canopy cover (based on low AIC_c_ weightings) in the present study (small-eyed snakes, velvet geckos, copper-tailed skinks and flat-rock spiders [21], [36]–[39]). The other species that did not respond to canopy cover in the present study (the red-throated skink) has not been studied experimentally in this respect (however, red-throated skinks prefer shadier habitat, potentially explaining this result [28]).

Why was the concordance between previous experiments and our field experiment excellent for one species (broad-headed snakes), and poor for the others? A likely reason is that we deployed all of the artificial rocks in open areas with high levels of incident radiation (canopy openness in our study ranged from 33–85%, canopy openness in nearby areas ranged from 15–75% [19]). The range in canopy openness above our artificial rocks was similar to that selected by reptiles in a previous field study at a nearby site (38–75% [19]). Thus, the areas where we deployed the artificial rocks largely provided optimal levels of canopy openness (and thus thermal regimes [19]), reducing the importance of thermal cues (and hence, elevating the relative importance of non-thermal cues) for retreat site selection. The relative importance of thermal *versus* other cues presumably differs among species, so that some taxa responded to temperature during our field trials whereas others did not.

Other macrohabitat correlates of faunal distribution are more difficult to interpret, and do not relate as closely to the parameters manipulated in previous experimental studies. The preference of copper-tailed skinks and red-throated skinks to use artificial rocks close to woodland (Table 1, Figure 2D) may reflect substrate attributes, because these lizards actively select rock-on-soil habitats (enabling burrow construction beneath the rocks) for nocturnal retreats [37], and soil depths typically are greater close to the woodland than on large open exposed areas of plateau. More puzzlingly, copper-tailed skinks also preferred rocks that were far from other rocks, and wall skinks preferred rocks that were far from leaf litter (Table 1, Figure 2F, 2G). The latter effect may reflect the predation risk posed by large invertebrates, such as centipedes and spiders [40], [41]; wall skinks are the smallest reptile species on these rock outcrops (mean snout-vent length 40 mm [42]), which may render them especially vulnerable to invertebrate predation [41].

### Frequency of Use of Artificial Rocks

Six species used individual artificial rocks frequently enough to allow comparisons within the subset of used artificial rocks (Table 2). Repeated use of individual artificial rocks by some taxa appears to be thermally driven. For example, the frequency of rock usage by two species (velvet geckos and flat rock spiders) was influenced by the amount of radiation received (Table 2), but other variables appear to influence these species differently. The tendency for velvet geckos to reuse artificial rocks located close to woodland may indicate a preference for proximity to foraging sites, and the reuse of artificial rocks located close to other rocks may reflect territoriality (these lizards often use two or three adjacent rocks as shelter and foraging sites [22], [36]). Interestingly, wall skinks showed an almost opposite trend to velvet geckos by repeatedly using artificial rocks located further from woodland, leaf litter and crevices (Table 2). As noted above, the small size of wall skinks may render them vulnerable to large invertebrate (centipedes, scorpions [40], [41]) and vertebrate (small-eyed snakes [43]) predators that forage in these areas. Flat rock spiders repeatedly used artificial rocks located close to other rocks, far from leaf litter and far from crevices (Table 2). These spiders are sedentary cannibalistic predators [44], and are vulnerable to larger invertebrates (such as huntsman spiders, *Sparassidae* spp., and large centipedes) that forage in these areas. Small-eyed snakes are ecological generalists [43] and in our study, showed few strong landscape-scale preferences in terms of which rocks they used repeatedly (Table 2). These snakes may readily shelter under any rocks that provide broadly suitable crevice dimensions, thermal regimes and foraging opportunities (leaf litter).

In addition to suggesting novel hypotheses about cues for macrohabitat selection in our study species, our results have direct implications for conservation and management of this system. Given the highly endangered status of the broad-headed snake [45], [46], the macrohabitat correlates of its retreat site selection are of particular interest. A trend for these snakes to be most abundant in sites close to steep cliffs has long been noted [46], [47], but has been attributed to the role of these cliffs in creating canopy gaps that allow solar radiation to warm the rocks [7], [19], [30]. Laboratory experiments and experimental field studies also have demonstrated that thermal regimes beneath rocks influence rock selection by the snakes [19], [28], [38]. However, our data suggest that proximity to cliffs also has a different and more direct effect on broad-headed snakes, perhaps by facilitating escape because these snakes readily escape over the cliff edge when we attempt to capture them (BMC pers. obs).

More generally, our results can guide attempts at habitat restoration in such a system by identifying how alternative manipulations are likely to affect target species. To enhance habitat suitability for the endangered broad-headed snake, for example, special effort should be given to creating suitable retreat sites close to cliff edges (for the snakes) and in areas exposed to high levels of solar radiation (for velvet geckos, a major prey species for the snake [48]). Future work could usefully explore the functional significance of macrohabitat-scale factors for the fitness (e.g., growth, survival) of individual reptiles, and hence clarify why the species that we studied differ so profoundly in the landscape features that predict their spatial distribution (Tables 1 and 2, Figure 2). Integrating information on criteria for habitat selection at a range of spatial scales can substantially improve our understanding of the determinants of spatial distribution of these animals. Ongoing landscape modification such as bush-rock removal (eliminating a non-renewable critical habitat), alteration of forest cover and climate change (and potentially, their interactions) threaten this rock dwelling faunal assemblage [13], [19], [25], [28], [30], creating a special urgency in understanding how artificial retreat sites can be used to mitigate these effects.

Retreat site selection by fauna is of great interest in many systems [49]–[51]. Future research could benefit by standardizing attributes of retreat sites that are important determinants of faunal use. For example, artificial retreat sites often are used to assist in the capture of elusive animals [52]–[54], restoration of degraded systems [27], [55], [56] and increased productivity of animal populations harvested for human consumption or use [57]. By standardizing retreat sites to account for factors that influence faunal use, and monitoring their subsequent usage by animals in the field, we may discover less obvious, but perhaps equally important, determinants of retreat site selection. In turn, a better understanding of the factors affecting the spatial distribution of animals across the landscape can facilitate management and conservation.
